# N-Cadherin-Mediated Epithelial–Mesenchymal Transition as a Prognostic Indicator in Canine Melanoma

**DOI:** 10.3390/vetsci12111023

**Published:** 2025-10-22

**Authors:** Thacyana Beatriz Guimarães Lopes, Daiana Yively Osorio Taborda, Clarice Soares Fenelon, Nayara de Oliveira Duarte, Karen Yumi Ribeiro Nakagaki, Camila Costa Abreu, Mayra Cunha Flecher, Helen Lima Del Puerto, Enio Ferreira

**Affiliations:** 1 Departamento de Patologia Geral, Universidade Federal de Minas Gerais, Belo Horizonte 31270-901, MG, Brazil; thacyanalopes29@gmail.com (T.B.G.L.); daosoriomvz@gmail.com (D.Y.O.T.); claricefenelon@gmail.com (C.S.F.); nayarad1@hotmail.com (N.d.O.D.); helendelpuerto@hotmail.com (H.L.D.P.); 2 CelulaVet—Centro de Diagnóstico Veterinário, Belo Horizonte 31365-000, MG, Brazil; karenyumi@ymail.com; 3 PatoVetVale—Patologia Veterinária do Vale, Taubaté 12050-470, SP, Brazil; camilacabreu@gmail.com; 4 Faculdade de Medicina Veterinária, Universidade Vila Velha, Vila Velha 29102-623, ES, Brazil; mayra.flecher@uvv.br

**Keywords:** melanoma, metastasis, survival analysis, cadherins, canine, epithelial-mesenchymal transition

## Abstract

**Simple Summary:**

Canine melanoma is a clinically significant neoplasm with important prognostic implications. This study evaluated E-cadherin and N-cadherin expression in 32 primary canine melanomas and their regional lymph nodes. Among these samples, 17 were metastatic and 15 were non-metastatic. Regional lymph node metastases were detected in 53.2% (17/32) of cases. High N-cadherin expression in primary tumors was associated with metastasis and reduced survival, whereas E-cadherin expression did not differ between metastatic and non-metastatic tumors. Metastatic primary tumors showed a positive correlation between E- and N-cadherin expression. These findings suggest that N-cadherin could serve as a prognostic marker and underscore the potential of targeting the Epithelial–mesenchymal transition (EMT) as a strategy to control metastasis.

**Abstract:**

Canine melanoma is a relatively common and clinically significant neoplasm in veterinary oncology, with important prognostic implications. Epithelial–mesenchymal transition (EMT), associated with changes in cell adhesion and increased metastatic potential, remains poorly characterized in this tumor type. We evaluated E-cadherin and N-cadherin expression in 32 primary canine melanomas and their regional lymph nodes, assessing associations with tumor progression and survival. Regional lymph node metastases were observed in 53% of cases. High N-cadherin expression in primary tumors correlated with metastasis and reduced survival, while E-cadherin expression showed no difference between metastatic and non-metastatic tumors. Notably, metastatic primary tumors exhibited a positive correlation between E- and N-cadherin expression. These results indicate that N-cadherin is a potential prognostic marker; and highlight the need for further studies on EMT as a therapeutic target to control metastasis.

## 1. Introduction

Melanocytic tumors account for approximately 7% of all malignant neoplasms in dogs and are recognized as among the most aggressive cancers due to their high metastatic potential [1]. These tumors predominantly affect the oral cavity, skin, sublingual region, and mucocutaneous junctions [2,3,4]. Canine oral melanomas, in particular, are frequently diagnosed at advanced stages and are associated with a high prevalence of pulmonary metastases, ranging from 17% to 51% [4].

As observed in multiple malignancies, melanoma invasion and progression are closely linked to alterations in cell adhesion, which play a pivotal role in tumor invasiveness, tumor–stroma interactions, and intracellular signaling pathways [5]. These processes are often mediated by the epithelial–mesenchymal transition (EMT), a critical mechanism underlying tumor dissemination [6]. EMT is characterized by the phenotypic conversion from an epithelial to a mesenchymal state, accompanied by substantial changes in cadherin expression profiles within neoplastic cells [7]. Specifically, epithelial adhesion molecules such as E-cadherin are downregulated, whereas mesenchymal markers, particularly N-cadherin, are upregulated. This molecular reprogramming confers enhanced migratory and invasive capacities, increased resistance to apoptosis, and the ability to produce extracellular matrix (ECM) components [6,8,9].

The adoption of a mesenchymal phenotype by neoplastic melanocytes is closely associated with tumor invasiveness and metastatic potential, as these cells lose epithelial contacts and preferentially adhere to fibroblasts and ECM proteins [10]. In this context, N-cadherin *(CDH2)* is frequently overexpressed in neoplastic cells and has been implicated in facilitating tumor progression [8,11].

Although EMT has been extensively documented in both canine and human tumors, including melanomas, its prognostic significance in dogs remains incompletely understood [12]. Therefore, the present study aims to comprehensively characterize cadherin expression associated with EMT in primary canine melanomas and their corresponding metastatic lymph nodes, and to evaluate their potential correlation with overall survival in affected animals.

## 2. Materials and Methods

### 2.1. Ethical Aspects

This work was carried out following the ethical principles for using animals in experimentation and after approval by the Animal Experimentation Ethics Committee (CEUA) of the Federal University of Minas Gerais (Protocol 228/2023).

### 2.2. Cases

Formalin-fixed, paraffin-embedded tissue specimens of melanomas were collected from the archives of the laboratory, obtained from neoplastic lesions surgically excised at different clinics in Brazil. The samples were collected between 2015 and 2024. The dataset comprised 32 primary melanomas and their corresponding regional lymph nodes, 17 of which (53.2%) showed histological evidence of metastasis. Clinical follow-up extended up to 20 months after primary excision.

Cases were included only after histological and immunohistochemical (Melan-A, and Melanoma Antigen) confirmation of melanoma in both primary tumors and regional lymph nodes. Inclusion criteria comprised the absence of chronic comorbidities that could affect survival assessment, availability of the primary tumor and regional lymph nodes for histopathological analysis, no chemotherapy before or after diagnosis, and confirmed death information provided by the attending veterinarian. The presence of a second neoplasm in the same animal was considered an exclusion criterion.

### 2.3. Histopathological Evaluation

Histological sections of 4 µm of tissues, stained with the hematoxylin-eosin (HE) technique, were re-examined to confirm the morphological diagnosis, according to the criteria established for diagnostic confirmation of melanoma. The following characteristics were determined in the histopathological evaluation: the morphology of neoplastic cells (spindle or epithelioid), nuclear atypia, pigmentation of the lesions, evidence of vascular invasion (neoplastic embolus), ulceration, desmoplasia, necrosis and presence of junctional activity (lentiginous or pagetoid) [12].

### 2.4. Immunohistochemistry

Samples containing preserved tumor tissue, with minimal areas of necrosis, ulceration, or intense inflammation, were included for analysis. 4 µm thin sections of selected tissue blocks were cut, mounted on gelatinized glass slides, and dried at 38 ° C overnight. Each tumor was evaluated as a whole, without differentiating between peripheral and central regions. The immunohistoperoxidase method with identification from polymerized secondary antibodies was used in this procedure. Antigen retrieval was carried out using pressurized heat at a temperature of 125 °C for 40 min (Pascal^®^, Agilent Technologies, Carpinteria, CA, USA), with a citrate solution, pH 6.0 (DakoCytomation Target Retrieval Solution, Agilent Technologies, Glostrup, Denmark), or ethylenediaminetetraacetic acid (EDTA), pH 9.0 (DakoCytomation Target Retrieval Solution), diluted to a concentration of 10%, and then cooled to room temperature for 20 min. The slides were incubated in a 3% H_2_O_2_ solution in methanol to block endogenous peroxidase activity. All reagents were applied manually. The primary antibody was incubated overnight (16 h) at 8 °C, while the remaining reagents were incubated for 30 min each, except for the chromogen 3,3′-Diaminobenzidine (DAB substrate system, DakoCytomation), which was applied for 3 min. Slides were counterstained with Giemsa and Harris hematoxylin to allow differentiation of melanin pigmentation into green. As positive controls, canine cutaneous tissue was used for E-cadherin, Melan-A, and Melanoma Antigen, and brain tissue was used for N-cadherin. Negative controls were prepared by replacing the primary antibody with standard mouse serum. Details of the primary antibodies, including their sources, clones, and key reagents, are provided in Table 1.

### 2.5. Immunohistochemistry Assessment

All histological analyses were performed using conventional optical microscopy (Olympus BX41, Olympus Corporation – Tokio - Japan ). Immunohistochemical expression was evaluated by a single pathologist (E.F.) with over 20 years of experience. Positivity for Melan-A and Melanoma Antigen was determined by cytoplasmic staining of a distinct brownish color in neoplastic cells, assessed according to a previously described semi-quantitative method: cases presenting cytoplasmic staining in more than 10% of neoplastic cells were considered positive [12]. Membranous, cytoplasmic, and nuclear labeling of N-cadherin, as well as membranous labeling of E-cadherin in neoplastic cells, were assessed and classified according to the percentage of labeled cells: 0 (no labeling), 1 (<25%); 2 (25–50); 3 (50–75%); and 4 (>75%). Tumors with scores of 3 or 4 were considered to exhibit high expression of the studied proteins [9].

Histological and immunohistochemical images were captured using an Olympus BX43 microscope equipped with a QColor 5 digital camera. Image acquisition was performed with QCapture Pro^®^ software, version 6.0 (QImaging, Rochester, NY, USA). All images were obtained under consistent lighting and magnification conditions.

### 2.6. Survival Time

Survival time was defined as the period (months) between surgical tumor removal and date of death caused by the disease (end point). Animals were excluded from the analysis if they were not monitored. A total of 13 animals with melanomas were assessed in a 20 month follow-up. The cause of death was confirmed at post-mortem examination. Animals that were alive and that died of unknown causes or causes unrelated to the tumor were considered censored.

### 2.7. Statistical Analysis

The Graphpad Prism v. 8.0.2 (GraphPad, San Diego, CA, USA), was used for statistical analysis. To analyze these categorized parameters, the association of variables was evaluated using the Chi-square test, and the correlation using the Spearman test. The survival curves were calculated using the Kaplan-Meyer estimate. Using the log-rank test, values were considered statistically significant when *p* < 0.05.

## 3. Results

### 3.1. Clinical-Histopathological Parameters

All 32 cases included lymph nodes, which were analyzed for the presence or absence of metastasis. Of the 32 cases evaluated, 46.8% (15/32) were classified as non-metastatic and 53.2% (17/32) as metastatic. Oral melanomas comprised 59% (19/32) of the cohort, including 10 metastatic and 9 non-metastatic cases, while cutaneous melanomas accounted for 41% (13/32), with 7 metastatic and 6 non-metastatic cases. The distribution of histological characteristics is summarized in Table 2, and the chi-square test did not reveal statistical significance.

### 3.2. Immunohistochemical Evaluation

**Figure 1:**
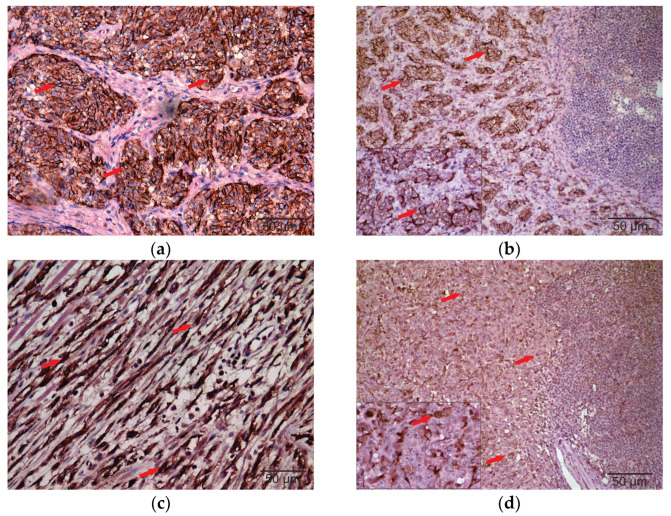
Immunohistochemistry of E-cadherin and N-cadherin in primary tumors and regional metastatic lymph nodes of canine oral melanomas. Images (**a**) and (**c**) were captured at 40× magnification. Images (**b**) and (**d**) are composite panels: the main images were captured at 20× magnification, and the inset images were captured at 40× magnification to highlight specific areas of interest. Some panels were assembled from adjacent microscopic fields to enhance visualization of immunostaining patterns. (**a**) Strong membranous staining of E-cadherin in the primary tumor. (**b**) Moderate membranous staining of E-cadherin in the regional metastatic lymph node. (**c**) Strong nuclear and cytoplasmic staining of N-cadherin in the primary tumor. (**d**) Weak nuclear and cytoplasmic staining of N-cadherin in the regional metastatic lymph node. Red arrows indicate areas of specific immunolabeling. Scale bars are included in all images.

Melan-A and PNL-2 immunoreactivity was detected as brown cytoplasmic staining in all samples, confirming the diagnosis of melanoma. E-cadherin showed membranous immunoexpression, ranging from weak to moderate, in 93.75% (30/32) of cases. All 32 cases exhibited cytoplasmic and nuclear N-cadherin immunostaining in neoplastic melanocytes, with moderate to strong intensity (Figure 1). These findings confirm the expression patterns of both cadherins in canine melanomas.

**Figure 2:**
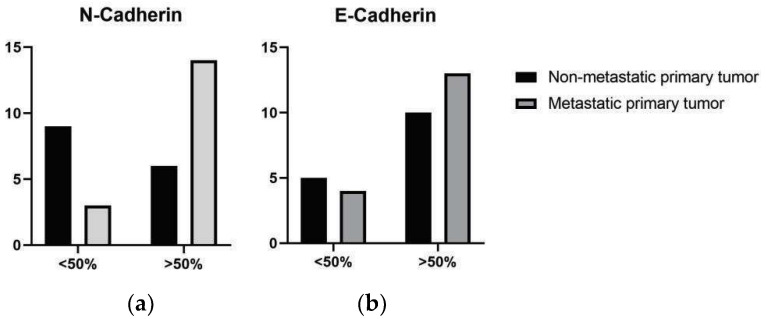
Frequency of E-cadherin and N-cadherin expression in non-metastatic and metastatic primary tumors. (**a**) Semi-quantitative score of N-cadherin expression, classified as low expression (50% immunolabeled cells; scores 3 and 4) (*p* = 0.0269). (**b**) Semi-quantitative score of E-cadherin expression, classified as low expression (50% immunolabeled cells; scores 3 and 4) (*p* = 0.6989).

Metastatic melanomas exhibited a higher proportion of cases with elevated N-cadherin immunostaining scores (scores 3 and 4) in 14/17 cases (43.75%), compared to non-metastatic melanomas in 3/15 cases (18.75%), with a significant difference between the groups (*p* = 0.0269) (Figure 2). When stratified by primary site (oral vs. cutaneous), no significant differences in N-cadherin staining scores were observed between metastatic and nonmetastatic tumors (Table 3). At metastatic sites, N-cadherin expression was most frequently observed with scores 3 and 4 (8/17 cases), followed by score 1 (7/17 cases).

Regarding E-cadherin expression, a high proportion of cases exhibited elevated staining scores (scores 3 and 4) in both metastatic tumors (56.52%, 13 cases) and non-metastatic tumors (43.48%, 4 cases), with no significant difference between the groups (*p* = 0.6989) (Table 3). Similarly, in metastatic lymph nodes, E-cadherin expression was most frequently scored as 3 (4/17 cases) and 4 (9/17 cases) (Table 3).

In metastatic melanomas, tumors with high N-cadherin expression scores also exhibited high E-cadherin expression scores, with a significant positive correlation between these expressions (r = 0.539; *p* = 0.025). This correlation (N-cadherin x E-cadherin) was not observed in non-metastatic melanomas (r = 0.037; *p* = 0.895) or in lymph node metastases (r = 0.247; *p* = 0.339). Although a high frequency of cadherin expression was observed in metastatic tumors and lymph node metastases, no significant correlation was found between these proteins in primary metastatic tumors and their corresponding lymph node metastases (E-cadherin: r = 0.253; *p* = 0.327; N-cadherin: r = 0.089; *p* = 0.733).

Additionally, tumors were analyzed according to the primary site (oral vs. cutaneous). In non-metastatic oral lesions, a positive correlation was observed between E-cadherin and N-cadherin expression (r = 0.684; *p* = 0.042), which was not observed in metastatic melanomas. In non-metastatic cutaneous lesions, a negative correlation between the expression of these proteins was found (r = −0.926; *p* = 0.008). Interestingly, in metastatic cutaneous melanomas, this correlation was reversed, showing a significant positive correlation between E-cadherin and N-cadherin expression (r = 0.776; *p* = 0.040).

### 3.3. Survival Time

**Figure 3:**
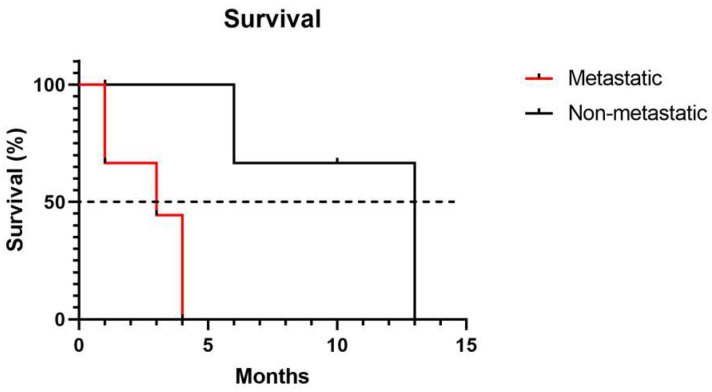
Survival analysis in dogs with non-metastatic and metastatic melanomas.

Animals with primary metastatic tumors exhibited a significantly shorter survival time, reaching the median at 3 months, compared to animals with non-metastatic melanomas, which reached the median at 13 months (*p* = 0.0141) (Figure 3).

**Figure 4:**
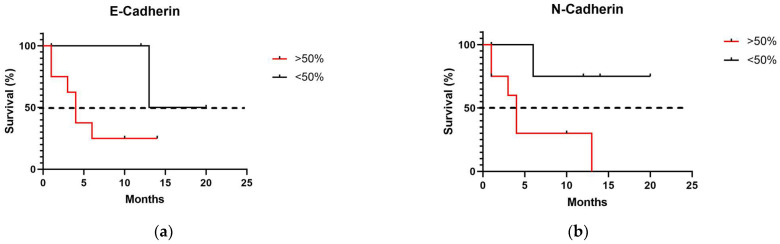
Survival analysis in dogs with melanoma according to cadherin expression, considering low expression, 50% (score 3 and 4): (**a**) E-cadherin; (**b**) N-cadherin.

Considering cadherin immunoexpression in all primary melanomas studied, tumors with high N-cadherin expression scores (3 and 4) exhibited a significantly shorter survival time, with a median of 4 months, compared to tumors with low expression scores (0, 1, and 2) (*p* = 0.0410). Regarding E-cadherin expression, no significant differences were observed between tumors with high and low expression (Figure 4).

## 4. Discussion

The prognosis of dogs with melanomas remains difficult to predict using standard parameters, such as histological type, invasion indices, or proliferation rates, highlighting the need for additional prognostic markers [12]. In this study, aberrant N-cadherin expression emerged as a potential prognostic biomarker, being significantly associated with metastasis and reduced survival.

These findings corroborate previous evidence indicating that N-cadherin plays a pivotal role in EMT and tumor dissemination in melanomas [6,9,13]. Elevated N-cadherin expression facilitates melanoma cell adhesion to dermal fibroblasts and endothelial cells, enhances cell survival and migratory capacity, and activates anti-apoptotic pathways, including AKT/PKB, while upregulating catenin and inhibiting the pro-apoptotic factor BAD, thereby promoting tumor viability and metastatic potential [14].

Under physiological conditions, basal epidermal melanocytes express E-cadherin, regulating keratinocyte interactions and maintaining controlled proliferation. Several studies suggest that neoplastic melanocytes may lose E-cadherin expression and acquire a mesenchymal phenotype via EMT [15]. Interestingly, in the present study, high E-cadherin expression (>50%) was observed in both metastatic and non-metastatic melanomas, without statistically significant differences, consistent with previous reports [10].

Moreover, metastatic primary melanomas exhibited a significant positive correlation between N-cadherin and E-cadherin expression, suggesting a complex interplay during metastatic progression. This phenomenon may reflect a gain of N-cadherin without loss of E-cadherin expression, consistent with a partial EMT phenotype [16]. Circulating melanoma cells have also been reported to co-express E- and N-cadherin, representing this partial EMT state and a hybrid cadherin profile [6]. This hybrid epithelial–mesenchymal phenotype is associated with enhanced metastatic potential and poorer survival outcomes compared with cells undergoing a complete EMT program, consistent with findings previously reported in breast cancer [17].

Experimental evidence from murine breast cancer models demonstrates that cells exhibiting partial EMT possess higher pulmonary metastatic capacity than cells undergoing complete EMT [18]. This hybrid state is regulated by transcription factors including ZEB1, ZEB2, and Snail [19], as well as key signaling pathways such as TGF- and Wnt/-catenin, which jointly promote E- and N-cadherin co-expression [20]. Thus, the partial EMT observed in this study represents an important mechanism by which epithelial characteristics are retained while contributing to tumor aggressiveness and malignancy [9,21].

Analysis of metastatic lymph nodes revealed a lack of correlation between E- and N-cadherin expression, with a non-significant reduction in N-cadherin levels. This observation may reflect mesenchymal–epithelial transition, whereby neoplastic cells revert from a mesenchymal to an epithelial phenotype, facilitating adherence at the metastatic site [22].

Another key finding of this study is the confirmation of metastasis as a prognostic determinant in canine melanomas. Survival analysis indicated that animals with nonmetastatic tumors exhibited significantly longer survival compared with those with lymph node metastases, consistent with reports of poorer outcomes in metastatic cases [12].

Regarding the association of cadherin expression with survival, E-cadherin levels were not significantly correlated with survival outcomes. This finding aligns with Leme et al., who evaluated 89 colorectal adenocarcinoma patients and found no association between E-cadherin expression and tumor stage, recurrence, or survival [23]. Conversely, other studies have linked loss of E-cadherin to adverse prognosis and increased tumor aggressiveness [24,25].

In contrast, N-cadherin expression emerged as a robust prognostic indicator in canine melanoma, with high expression (>50%) correlating with reduced survival. Similar associations have been observed in human cancers: in breast cancer, N-cadherin expression enhances tumor cell motility and invasiveness [26], while in prostate cancer, elevated N-cadherin promotes cell motility, invasion, and metastasis [27]. In human melanomas, N-cadherin expression is generally increased in metastatic lesions compared to primary tumors, highlighting its role in epithelial-to-mesenchymal transition (EMT) and tumor progression [15]. Collectively, these data indicate that N-cadherin not only facilitates invasion and metastatic dissemination but also serves as a critical prognostic biomarker across multiple tumor types.

As with most retrospective studies in veterinary oncology, this work has certain limitations, primarily related to the relatively small number of cases and a 20-month follow-up, which may reduce the statistical power of correlation and survival analyses. However, similar sample sizes are frequently reported in the literature, as multicenter data collection and standardized follow-up remain challenging due to differences in diagnostic protocols and case availability [10,16].

Despite the limited sample size, the present findings provide meaningful pathological and molecular insights into canine melanomas, consistent with previous studies and supporting the continued development of prognostic and translational research in veterinary pathology. In particular, our study identifies N-cadherin expression by immunohistochemistry, offering evidence of its tissue localization and suggesting its involvement in tumor progression and prognostic significance. While immunohistochemistry reliably depicts protein distribution within tissues, complementary molecular techniques such as qPCR, Western blotting, or ELISA could further clarify expression levels and underlying mechanisms. Incorporating these approaches in future studies would help to validate and expand the findings reported here.

## 5. Conclusions

Metastasis in canine melanomas is strongly associated with high N-cadherin expression, indicating its role in promoting metastatic potential. Early diagnosis and management remain critical, and the complex patterns of E-cadherin and N-cadherin suggest partial epithelial–mesenchymal transition. These markers represent promising prognostic indicators and potential therapeutic targets in canine melanoma.

## Figures and Tables

**Table 1:** Antibodies and procedures used in the immunohistochemical study.

Antibody	Clone	Manufacturer Code	Manufacturer	Dilution	Antigen Retrieval Method	Secondary Antibody	Incubation Time
Melan-A	A103	SC-53537	Dako	1:100	Citrate + pressurized wetheat	Novolink *	16 h
Melanoma Antigen	PNL-2	SC-59306	SantaCruz	1:100	Citrate + pressurized wetheat	Novolink *	16 h
E-Cadherin	4A2C7	180223	Invitrogen	1:50	EDTA + pressurized wetheat	Novolink *	16 h
N-Cadherin	6G11	M3613	Dako	1:50	Citrate + pressurized wetheat	Novolink *	16 h

* Novolink Polymer Detection System; Leica Biosystems, Newcastle upon Tyne, UK; Agilent Technologies, Carpinteria, CA, USA; Santa Cruz, OR, USA; Invitrogen, Carlsbad, CA, USA; Dako, Carpinteria, CA, USA.

**Table 2:** Histopathological characteristics of the 32 cases included in the study.

n = 32	Metastatic Primary Tumor 17 (100%)	Non-Metastatic Primary Tumor 15 (100%)
Histological Type		
Epithelioid	9 (52.94%)	7 (46.67%)
Fusiform	2 (11.76%)	2 (13.33%)
Mixed	5 (29.41%)	6 (40.00%)
Balanoid	1 (5.88%)	0 (0%)
Degree of Pigmentation		
<50%	10 (31.25%)	7 (21.87%)
>50%	7 (21.87%)	8 (25.00%)
Necrosis		
Absent	1 (5.88%)	4 (26.67%)
Present	16 (94.12%)	11 (73.33%)
Lymphatic embolus		
Absent	5 (29.41%)	8 (53.33%)
Present	12 (70.59%)	7 (46.67%)
Atypia		
<20%	0 (0%)	0 (0%)
>20%	17 (100%)	15 (100%)
Ulceration		
Absent	0 (0%)	3 (20.00%)
Present	17 (100%)	12 (80.00%)
Desmoplasia		
Absent	4 (23.53%)	3 (20.00%)
Present	13 (76.47%)	12 (80.00%)

**Table 3:** Distribution of E-Cadherin and N-Cadherin expression in 32 cases of canine melanoma by location and metastatic status.

Location	Metastatic Status	Marker	High Expression n (%) *	Lower Expression n (%) *
Oral	Non-metastatic	E-Cadherin	6 (19%)	3 (9%)
		N-Cadherin	4 (13%)	5 (16%)
	Metastatic	E-Cadherin	9 (28%)	1 (3%)
		N-Cadherin	8 (25%)	2 (6%)
Cutaneous	Non-metastatic	E-Cadherin	4 (13%)	2 (6%)
		N-Cadherin	2 (6%)	4 (13%)
Cutaneous	Metastatic	E-Cadherin	4 (13%)	3 (13%)
		N-Cadherin	6 (19%)	1 (3%)

* High expression: score ≥3; Low expression: score ≤2.

## Data Availability

The original contributions presented in this study are included in the article. Further inquiries can be directed to the corresponding author.

## Abbreviations

The following abbreviations are used in this manuscript:

CAPES	Coordenação de Aperfeiçoamento de Pessoal de Nível Superior
CEUA	Animal Experimentation Ethics Committee
CNPQ	Conselho Nacional de Desenvolvimento Científico e Tecnológico
DAB	3,3-Diaminobenzidine
EDTA	Ethylenediaminetetraacetic acid
ECM	Extracellular matrix
EMT	Epithelial–mesenchymal transition
FAPEMIG	Fundação de Amparo à Pesquisa de Minas Gerais
HE	Hematoxylin-eosin
